# Progress in animal models for studying hyperuricemia

**DOI:** 10.3389/fphar.2025.1636205

**Published:** 2025-08-22

**Authors:** Senyue Zhang, Kaiqing Li, Hu Zhang, Tong Fu, Yanchun Ma, Shuxiang Zhang, Guoli Xing, Ying Tong

**Affiliations:** ^1^ Heilongjiang University of Chinese Medicine, Harbin, China; ^2^ Department of Rheumatology, Heilongjiang Academy of Traditional Chinese Medicine, Harbin, China; ^3^ Brandeis University, Waltham, MA, United States; ^4^ The First Affiliated Hospital of Heilongjiang University of Traditional Chinese Medicine, Harbin, China

**Keywords:** hyperuricemia, animal models, modeling method, gene editing, evaluation indicators

## Abstract

Hyperuricemia (HUA) is a prevalent metabolic disorder driven by dysregulated purine metabolism and impaired urate excretion, and robust animal models are critical for elucidating its pathophysiology and guiding therapy development. This review systematically examines chemically induced, gene‐edited, environmental, exercise and microbiota‐based HUA models across rodents, poultry, primates, zebrafish and silkworms, highlighting each model’s strengths and limitations in mimicking human uric acid handling. We discuss how these models have validated standard urate‐lowering treatments—such as xanthine oxidase inhibitors and uricosurics—and uncovered emerging therapeutic targets, including the gut–NLRP3 inflammasome axis and SIRT1‐mediated ABCG2 regulation. Finally, we propose a unified three‐tier framework encompassing biochemical, mechanistic and pathological criteria to standardize model evaluation and accelerate translational research in hyperuricemia.

## 1 Introduction

HUA is a chronic metabolic disease characterized by abnormal increase of serum UA level, and its pathogenesis is complex, which is mainly caused by purine metabolism disorder or uric acid excretion disorder (Liu et al., 2025). In recent years, the incidence of HUA has been on the rise all over the world, especially in China. According to research statistics, the overall prevalence rate of HUA in China has reached 17.7% (Du et al., 2024), and the prevalence rate of young men has reached 32.3%, showing an obvious trend of rejuvenation (Fang et al., 2023). HUA not only poses a new major threat to human health, but also is widely called the “fourth highest” after hypertension, hyperglycemia and hyperlipidemia because it is closely related to many chronic diseases (such as gout, kidney calculi’s disease, chronic kidney disease and cardiovascular disease) (Zhou P. et al., 2024). Therefore, it is of great significance to deeply explore the pathogenesis and prevention strategies of HUA for ensuring the life and health of residents.

**FIGURE 1:**
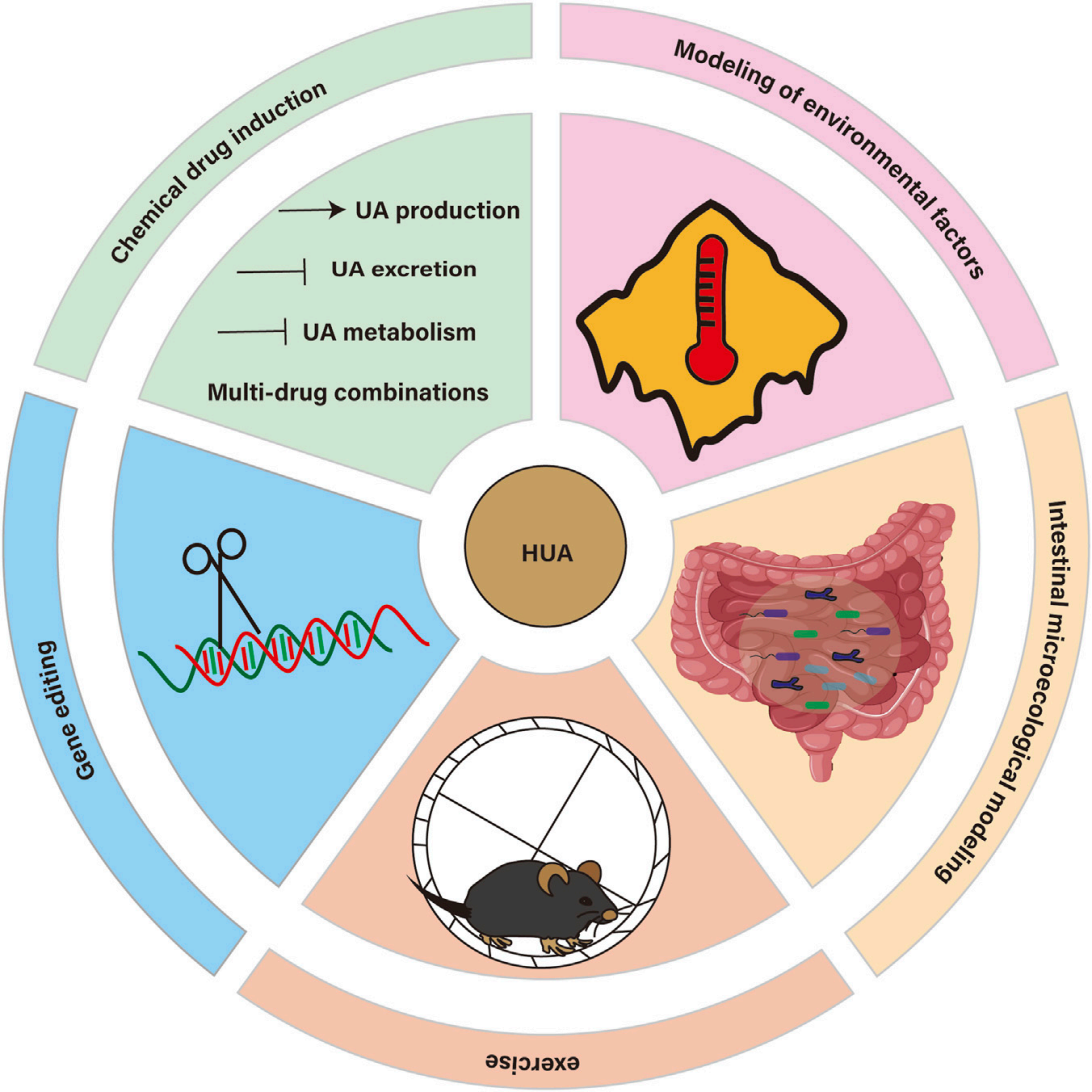
Overview of HUA’s modeling.

The pathogenesis of HUA is complex, which mainly comes from the excessive production and/or decreased excretion of UA in kidney and outside kidney, and is influenced by many factors such as heredity, environment and lifestyle (Nishizawa et al., 2022). UA is the final product of purine metabolism (Chang et al., 2025), and its sources include exogenous (dietary intake, accounting for about 20%) and endogenous (nucleic acid metabolism, accounting for about 80%) (Li K. et al., 2024). Excessive UA production is often due to high purine diet, accelerated nucleic acid metabolism or abnormal XOD activity (Yang et al., 2025); Decreased excretion is related to abnormal function of UA transporters in kidney and intestine (such as URAT1, GLUT9, ABCG2) or impaired renal function (Yanai et al., 2024). In addition, the excretion of UA mainly depends on the kidney (75%) and the intestine (25%), in which about 90% of the filtered UA will be reabsorbed by the renal tubules, and only a small amount will eventually be excreted, and this dynamic balance is regulated by UA transporters (Ohashi et al., 2023). Genetic factors affect the susceptibility of individuals to HUA through the polymorphism of UA metabolism-related genes (such as XOD, URAT1, GLUT9, ABCG2, UOX, etc.) (Liang et al., 2025). At the same time, environmental factors (such as high temperature and high humidity environment) (Wu Z. D. et al., 2022) and lifestyle high purine diet, high fructose intake, obesity, metabolic syndrome, etc.] (Kuwahara et al., 2017) will further interfere with UA metabolism. HUA can cause UA sodium salt crystals to deposit on joints, synovium and renal tubules, causing gouty arthritis and kidney calculi, increasing the risk of acute kidney injury and even developing into chronic kidney disease (Gu et al., 2025). Impaired renal function will further reduce UA excretion and form a vicious circle. Clinically, about 90% of HUA patients are caused by decreased renal excretion function (Richette and Bardin, 2010). See Figure 1. Soluble uric acid crystals activate TLR4 on podocytes, triggering MyD88-dependent NF-κB translocation and subsequent NLRP3 inflammasome assembly. This recruits ASC adaptors to activate caspase-1, which cleaves gasdermin D (GSDMD) to form plasma membrane pores. Concurrent IL-1β/IL-18 maturation and osmotic lysis drive podocyte pyroptosis, disrupting glomerular filtration barriers and manifesting as proteinuria.

In view of the complexity and diversity of HUA, establishing an ideal animal model of HUA is the basis for further studying its pathogenesis, screening drugs and developing treatment methods. The ideal animal model should be able to accurately simulate the pathological characteristics of human HUA, with good stability and repeatability, and be highly related to human diseases. At present, there are various methods to build animal models of HUA, including chemical drug induction (including increasing UA source, inhibiting UA excretion, inhibiting UA metabolism and multi-drug combination), gene editing, environmental factors modeling, exercise modeling and intestinal microecology modeling. These methods have their own advantages and limitations and are suitable for different research purposes and scenarios. Establishing the evaluation system of animal model is an effective standard to determine whether the model is successful, which has important scientific value and clinical significance for promoting the study of HUA. Therefore, this paper will systematically review the research progress of HUA animal models, including animal model screening, specific modeling methods and animal model evaluation system, in order to provide reference and guidance for the research of HUA.

To provide a more critical synthesis, we propose a three‐dimensional conceptual framework that maps each animal model onto its underlying pathogenic mechanism, modeling strategy and translational goal. Along the mechanistic axis, models are organized by whether they primarily recapitulate excess urate production, impaired excretion or inflammatory sequelae. Along the methodological axis, models are classified as chemical induction, gene editing, environmental/exercise challenge or microbiota modulation. Finally, the translational axis aligns each model with its contribution to drug validation, target discovery or biomarker development. By integrating these dimensions, this framework not only clarifies the rationale for choosing specific models but also highlights synergistic opportunities—such as combining gene‐edited and environmental approaches—to address complex facets of hyperuricemia and streamline the path from bench to bedside.

## 2 Screening of HUA animal models

### 2.1 Common types of animal models

#### 2.1.1 Characteristics and applications of rodents (rats, mice)

Rodents, such as rats and mice, are commonly used animal models in HUA research (Dong et al., 2023). They have the advantages of clear genetic background, strong reproductive ability, low feeding cost, and convenient experimental operation (Zeng et al., 2024), and their gene sequence and metabolic pathways are highly similar to humans, making them easy to observe and intervene in for a long time (Johnston et al., 2016). However, rodents have uricase genes in their bodies that can further break down uric acid into allantoin and excrete it from the body, which is different from the metabolic characteristics of humans lacking uricase, resulting in significant differences in their blood UA levels compared to humans. Therefore, in order to simulate the human HUA state, studies often use drugs such as potassium oxonate to inhibit uricase activity, or combine with other chemical agents to establish reliable HUA models. When selecting experimental animals, SD rats, Wistar rats, and Kunming DY rats are usually chosen, which are suitable for scenarios that require large tissue sample sizes, long-term experiments, complex surgical procedures, or behavioral studies (Gao et al., 2022); However, KM mice, ICR mice, and C57BL/6 mice are commonly used for mice. Among them, KM mice have a higher sensitivity to HUA induced drugs and are more suitable for establishing HUA models. Moreover, mice are more suitable for gene manipulation, high-throughput screening, or research utilizing the advantages of specific strains (Feng et al., 2024). In terms of gender selection, adult male rats or mice are usually preferred for modeling to ensure the stability and reproducibility of experimental results, as UA levels of female rodents are easily affected by hormonal fluctuations and vary greatly, and the body function of elderly mice is poor (Zhou H. et al., 2024). Although rodent models differ from humans in metabolic mechanisms, they remain the most commonly used animal models in HUA research due to their ease of management and similarity to human physiological and biochemical characteristics. They are widely used in basic research and drug screening fields.

Recent animal studies indicate clear sex differences in uric acid (UA) metabolism. Female rodents tend to be protected from hyperuricemia, likely due to estrogen. For example, high-fat diet–induced obese female mice developed steatosis and insulin resistance but no increase in serum UA (unlike males). Estrogen appears key: mature female rats have ∼40% lower hepatic xanthine oxidase (XO) activity (and UA production) than males, and ovariectomy restores XO activity to male levels. Estradiol treatment lowers serum UA by modulating transporters: it suppresses renal urate-reabsorptive transporters (URAT1, GLUT9) while upregulating the intestinal efflux transporter ABCG2 to enhance UA excretion. Consistent with this, estrogen-depleted (postmenopausal) models show higher UA: ovariectomized female rats on high-fructose/oxonate regimens had significantly higher serum urate and worse cardiometabolic outcomes than intact females. Aging also augments UA burden–older mice (and presumably rats) show higher serum UA, driven by stronger purine turnover and reduced clearance. In aged rodents, loss of renal excretory function (e.g., downregulation of OAT1/3, GLUT9) contributes to hyperuricemia, but interventions like butyrate or “young” gut microbiota can upregulate ABCG2 and OAT transporters and lower SUA. These insights have led to specific modeling recommendations: studies of postmenopausal or age-related HUA should employ female and/or older animals (for example, ovariectomized or ≥12–18-month rodents, often with purine-rich diets or uricase inhibitors) to mimic the estrogen-deficient or senescent state.

#### 2.1.2 Advantages and limitations of poultry animals (chickens, quails)

Poultry animals (chickens, quails) have unique advantages and limitations in HUA research. Its advantages are reflected in the lack of UA oxidase genes, and the purine metabolite UA is highly similar to human metabolic characteristics (Yan et al., 2024). The uric acid transporters BCRP and MRP4 are involved in excretion (Ding et al., 2019), and serum UA levels are regulated by GLUT9 (Ding et al., 2021). Dysregulation of these mechanisms may promote the progression of HUA. In pathological conditions, renal dysfunction can lead to an increase in serum uric acid and compensatory increases in BCRP and MRP4 in the ileum; However, under normal renal function, renal BCRP and MRP4 are the main regulatory factors for uric acid excretion. Therefore, poultry animals are suitable for high purine diet induction modeling, which can quickly induce pathological changes such as UA salt crystal deposition, simulating the pathological mechanism of human HUA. The breeding cost is low, the reproduction speed is fast, and the experimental period is short, making it suitable for rapid screening of UA lowering drugs (Yan et al., 2024). Having a larger body size can be used for blood collection and tissue sample collection, as well as for studying the effects of environmental factors and diet on UA metabolism (Wu Y. et al., 2022). However, poultry animals also have obvious limitations: they require a high feeding environment and need to maintain dry and well ventilated conditions. Damp, dark, and narrow spaces can easily lead to gout (Cole and Austic, 1980); Sensitivity to stress response may interfere with experimental results (Cedraz et al., 2017); The physiological structure (such as kidney function) differs greatly from mammals (K et al., 2024), and gene editing technology is relatively lagging behind, which limits the depth and breadth of gene level research (Lee et al., 2020). In addition, there is a gap between the microbial purification and detection technology of poultry animals and international research, and the feeding conditions are special, which makes it easy to spill feed and cause inaccurate inducers. These factors further limit their clinical application and transformation. In summary, although poultry animals have unique value in HUA research, they still face many challenges in clinical application and transformation due to species differences and difficult feeding and management, making it difficult to replace rodents as the mainstream HUA animal model in a short period of time.

#### 2.1.3 Exploration of other animal models (primates, zebrafish, silkworms)

Animal models such as primates, zebrafish and silkworm have unique advantages and broad prospects in the study of HUA. Primates are highly similar to humans in gene and physiological metabolism, and lack UA oxidase. After UA level rises, it is easy to have complications such as kidney damage and cardiovascular disease, which is consistent with the clinical manifestations of human HUA. Its immune system and nervous system are also very similar to human beings, and it is an ideal model to study the correlation between HUA and other systemic diseases. For example, taking inosine-induced acute HUA model in rhesus monkeys as an example (Tang et al., 2021), 200 mg/kg inosine significantly increased the blood UA level within 30 min (from 51.77 ± 14.48 μmol/L/L to 178.32 ± 14.47 μmol/L/L) and reached the peak value (201.41 ± 42.73 μmol/L/L) in 1 hour. However, primates have high feeding cost, long breeding cycle and limited application, and are mostly used to study the relationship between HUA and complex diseases such as cardiovascular disease and metabolic syndrome. As a new model organism, zebrafish has the advantages of transparent embryo, rapid development and *in vitro* fertilization, and its gene editing technology is mature, which can construct a variety of gene mutation models and is suitable for Qualcomm screening of UA-lowering drugs. The metabolic pathway of zebrafish UA is similar to that of human beings, and both lack UOX, which can simulate the pathological process of human UA accumulation. Its reproductive cycle is short, its egg production is large, its genome has high homology with human beings, its size is small, its maintenance cost is low, its main organs develop within 5–6 days after fertilization, and it can visualize and track cells, organs and tissues in real time (Zhang et al., 2019). The UA level of zebrafish larvae reached its peak on the fifth day after fertilization, and then decreased sharply due to the expression of Uox in the liver, so zebrafish embryos were used to construct the HUA model on the fifth day after fertilization (Deng et al., 2024). However, zebrafish is different from humans in evolution, physiology and metabolism, so it needs to be combined with other models for comprehensive research. As an important model insect, the complete genome sequence of silkworm has been determined, its genes can be edited, and many mutant lines have been constructed, which can increase UA level by knocking out UXO gene and simulate the pathological characteristics of human HUA (Orhan and Deniz, 2021). The metabolism of purine in silkworm and human is similar, and both of them do not express UA enzyme. XOD, as a key enzyme, catalyzes the oxidation of hypoxanthine to xanthine and further catalyzes it to UA. The experiment showed that (Zhang et al., 2012), the XOD activity in hemolymph and UA concentration in hemolymph and fat decreased in a dose-dependent manner, indicating that the dependence of UA metabolism in silkworm on XOD was similar to that of human beings. *Bombyx mori* UA mainly originates from fat body, and is excreted through Malpighian tubule-hindgut system (Pan et al., 2017). The synthesis and excretion ability of UA changes at different development stages. The level of UA in hemolymph and fat body of fifth instar larvae is the lowest, and its excretion and storage control mechanism is similar to that of tobacco hornworm (Lismont et al., 2015). Therefore, its regulation in UA metabolism and the pathogenesis of HUA can be further studied. In addition, the silkworm is large and can separate organs such as midgut, which is convenient for studying the metabolism and drug effects of UA in organs. Moreover, it has low feeding cost and rapid reproduction, and can provide a large number of experimental samples without ethical and biological safety hazards. To sum up, silkworm as a HUA model has a solid theoretical basis, and its application potential will be more extensive and in-depth with the development of gene editing and experimental technology. The interspecies heterogeneity in UA metabolism necessitates model-specific evaluation standards, which we synthesize in Table 1. This framework cross-references uricase status, dominant excretion pathways, and human disease relevance to align biomarker selection with physiological fidelity.

**TABLE 1:** Cross-species standardization framework for hyperuricemia evaluation.

Model	Uricase status	Dominant UA excretion pathway	Core assessment metrics	Human relevance
Rodents	Functional	Renal (70%)	Serum UA↑ + Urinary allantoin↓ + Renal URAT1↑ + Hepatic XOD↑	Moderate (★★☆)
Poultry	Absent	Intestinal (60%)	Serum UA↑ + Articular crystals + Plasma BCRP↓	High (★★★)
Primates	Absent	Renal (75%)	Serum UA↑ + Renal KIM-1↑ + Cardiovascular markers	Very High (★★★★)
Zebrafish	Embryonic-only	Branchial/Intestinal	Larval UA↑ + Oxidative stress (MDA↑) + gill ABCG2a↓	Moderate (★★☆)
Silkworms	Absent	Malpighian tubules	Hemolymph UA↑ + Fat body XOD↑ + Growth retardation	Low (★★☆)

Human Relevance Score: ★★☆ = moderate, ★★★ = high, ★★★★ = very high.

### 2.2 HUA animal selection factors

**FIGURE 2:**
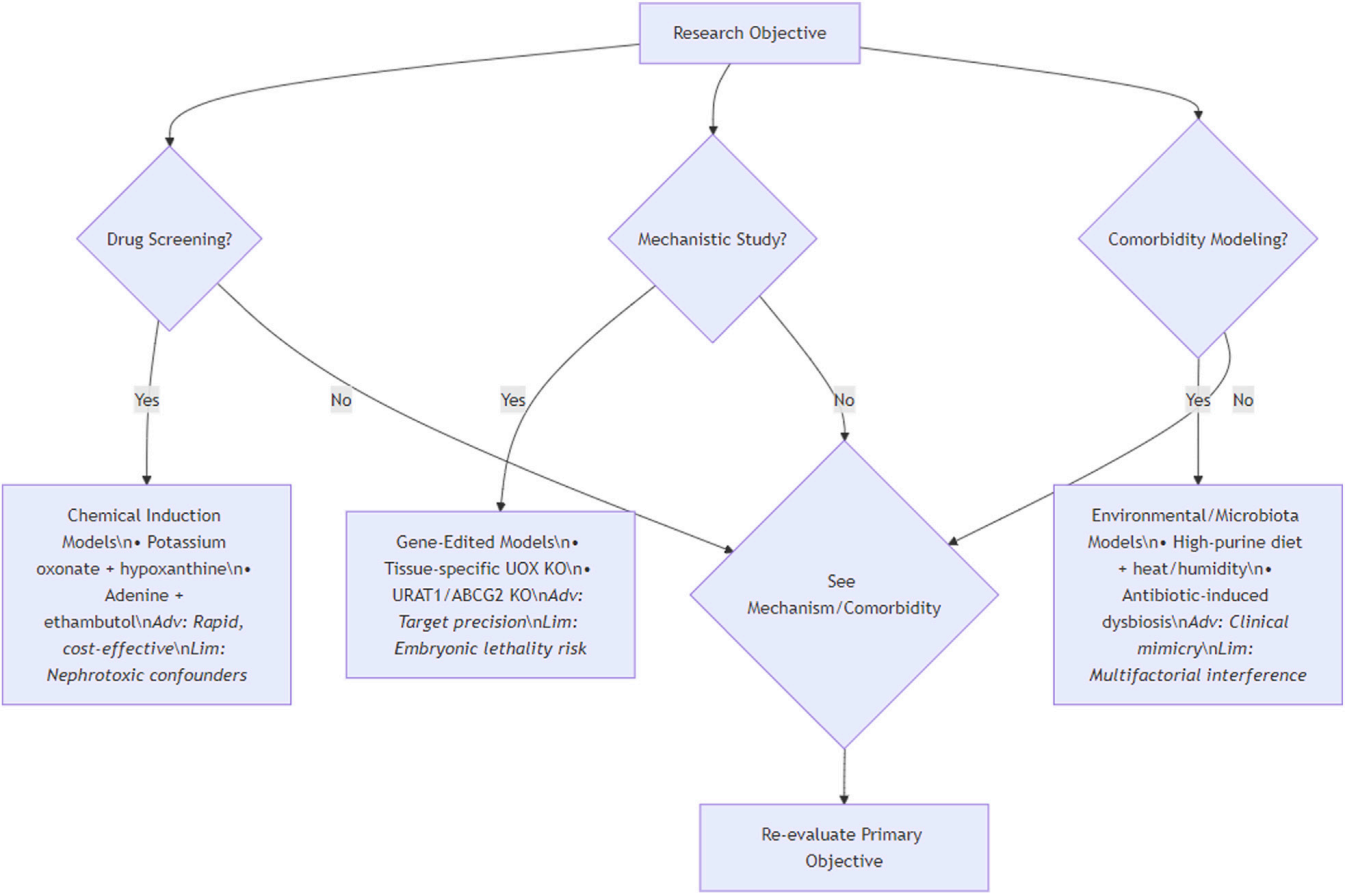
Decision flowchart for selecting hyperuricemia animal models.

In the study of HUA, the choice of animal model is very important. The differences in metabolic characteristics, physiological structure and genetic background of different species of animals determine their accuracy in simulating the pathological state of human HUA. Although primates are highly similar to humans, they are limited by feeding costs, reproductive cycle and ethical constraints. The gene sequence and metabolic pathway of rodents are similar to those of humans, which is convenient for long-term observation. Different strains have different responses to induced drugs, which is beneficial to the study of genetic susceptibility. Birds lack UXO, and the blood UA level increases significantly after high purine diet, which is suitable for large-scale experiments, but the physiological structure is quite different from that of mammals. Emerging models such as zebrafish and silkworm have their own advantages, but their applications are still in the initial stage. When designing the experiment, the individual characteristics of animals, such as age, sex, weight and health status, have a significant impact of UA metabolism on renal injury, which should be fully considered. At the same time, feeding management conditions and costs are also important considerations. Researchers need to synthesize these factors and accurately select the most suitable animal model according to the research purpose and experimental conditions, so as to ensure the scientificity, reliability and transferability of the research results and provide strong support for the research and treatment of HUA. To standardize model selection, Figure 2 provides a decision workflow: For drug screening, prioritize chemically induced models (e.g., oxonate-hypoxanthine) offering rapid UA elevation; for mechanistic studies, gene-edited systems (e.g., tissue-specific UOX KO) enable target validation; for comorbidity research, environmental/microbiota models (e.g., high-purine diet with heat stress) best replicate clinical synergies. This protocol balances physiological fidelity and practicality.

## 3 Molding method of HUA

The main routes of administration in animal models include feeding, gavage, subcutaneous injection, intraperitoneal injection and gene knockout. Among them, avian species are usually fed, knockout technology is mostly applied to mice, while primates, zebrafish and silkworms are mainly fed and gavaged. Rats and mice, on the other hand, cover all these modes of administration. According to the mechanism of HUA formation, there are five main methods for preparing animal models of HUA: chemical-induced models (increasing UA source, inhibiting UA excretion, inhibiting UA metabolism, and multi-drug combination modeling), gene editing models, environmental factor modeling, exercise modeling, and microecological modeling. The specific data are shown in Tables 2, 3.

**TABLE 2:** Simplified chemically induced hyperuricemia models.

Induction type	Model	Core method	Primary outcomes
Increased UA source	Adenine	Oral/Gavage administration	Serum UA↑, Renal crystals
Increased UA source	Hypoxanthine	Intraperitoneal injection	Serum UA↑, Hepatic XOD↑
Increased UA source	Yeast extract	Dietary supplementation	Serum UA↑, Articular deposits
Increased UA source	Fructose	Liquid diet supplementation	Serum UA↑, Intestinal ABCG2↓
Inhibited UA excretion	Ethambutol	Combined gavage with adenine	Serum UA↑, Tubular injury
Inhibited UA excretion	Niacin	Dietary modification	Hemolymph UA↑
Inhibited UA metabolism	Potassium oxonate	Intraperitoneal injection	Serum UA↑, Renal edema
Multi-drug combination	Oxonate + Hypoxanthine	Co-administration	Serum UA↑, Urinary UA↓

Chemically induced hyperuricemia models: Classification by induction mechanism (increased uric acid sources, inhibited excretion, inhibited metabolism, multi-drug combinations), with corresponding model organisms, core methods, and primary biochemical/pathological outcomes.

**TABLE 3:** Simplified non-chemical hyperuricemia models.

Model type	Specific approach	Core method	Primary outcomes
Gene editing	UOX knockout	CRISPR/Cas9 modification	Serum UA↑, Metabolic dysregulation
Gene editing	GLUT9 knockout	Tissue-specific deletion	Serum UA↑, Fetal developmental defects
Gene editing	URAT1 knockout	Humanized gene replacement	Serum UA↑, Altered UA/creatinine ratio
Gene editing	ABCG2 knockout	Systemic knockout	Serum UA↑, Intestinal excretion↓
Environmental factors	High-temperature	Controlled climate exposure	Serum UA↑, Renal URAT1↑
Exercise intervention	Forced treadmill	High-intensity protocol	Serum UA↑, Neutrophil infiltration
Intestinal microbiota	Antibiotic dysbiosis	Broad-spectrum treatment	Serum UA↑, Altered Firmicutes ratio

Non-chemical hyperuricemia models: Gene-edited (UOX, GLUT9, URAT1, ABCG2 knockouts), environmental (high-temperature exposure), exercise (forced treadmill), and microbiota-based (antibiotic dysbiosis) approaches, detailing specific methods and key phenotypic outcomes.

### 3.1 Modeling of chemically induced HUA

#### 3.1.1 Increased access to UA sources

##### 3.1.1.1 Construction of the adenine induction model

Adenine is a precursor substance for UA production, which is catalyzed by adenine phosphoribosyltransferase (APRT) *in vivo* (Silva et al., 2008) and combines with phosphoribosyl pyrophosphate (PRPP) to produce adenine nucleotide (AMP) (Norambuena et al., 2023). Excessive intake of adenine results in a large increase in the production of AMP, which is deaminated by adenylate deaminase (ADA) and converted to inosine monophosphate (IMP) (Li et al., 2017). IMP is oxidized to xanthine monophosphate (XMP) by inosine monophosphate dehydrogenase (IMPDH), which is subsequently converted to xanthine (Xan) by guanine monophosphate deaminase (GMPD) (Roy et al., 2016). Finally, Xan is oxidized to UA under the catalytic action of XOD, and since the amount of UA generated far exceeds the metabolic transformation and excretion capacity of the body, a large amount of UA cannot be excreted in time, resulting in a significant increase in the concentration of UA in the blood, and thus a model of adenine-induced HUA has been constructed. ZhangD et al. induced a model of HUA in male KM mice by adenine at 75 mg/kg for 28 days, and the results showed that the serum uric acid level was significantly higher in the modeling group of mice compared to the control group (737.22 ± 98.65 μmol/L vs 307.00 ± 56.61 μmol/L, *P* < 0.05) (Zhang et al., 2018). YangL et al. successfully induced HUA in 8-week-old or 12-week-old male C57BL/6J mice by feeding them diets containing different concentrations of adenine (0.3%, 0.2%, and 0.15%) for 4 weeks 6J mice for 4 weeks and successfully established a HUA model. The results showed that the serum UA levels of the mice in the model group increased significantly from 92.9 ± 10.1 μmol/L to 217.4 ± 18.6 μmol/L, indicating that the adenine diet-induced HUA model was successfully constructed (Yang et al., 2023). Adenine elevates UA but concurrently causes renal crystal obstruction, limiting its utility to HN studies. Urate-specific investigations should employ fructose or genetic models.

##### 3.1.1.2 Construction of a model for hypoxanthine induction

Hypoxanthine is a direct precursor for UA production. *In vivo* hypoxanthine is catalyzed by XOD to generate UA and produces ROS as a byproduct. When the increase in UA production exceeds the renal excretion capacity, the serum UA level rises, inducing HUA. ROS can induce oxidative stress, which disrupts the function and expression of the renal uric acid transporter protein, leading to enhanced renal reabsorption and decreased secretion of UA, thus exacerbating HUA. NiuY et al. successfully established a model of HUA by a single intraperitoneal injection of hypoxanthine at a level of 100 mg/kg. One hour after hypoxanthine injection, the serum UA level of mice increased significantly from the basal value of 135.0 ± 25.0 μmol/L to 224.5 ± 48.1 μmol/L, suggesting that the HUA model was successfully constructed (Niu et al., 2018). GuoZ et al. successfully constructed a HUA model by administering 300 mg/kg hypoxanthine intraperitoneally to 8-week-old C57BL/6 mice and subcutaneous injection of 100 mg/kg potassium ethoxyphenoxypropionate for 14 consecutive days, and successfully constructed a HUA model. The results showed that serum UA, urea nitrogen, creatinine levels and 24-h uric acid excretion were significantly increased in the model mice, hepatic xantho XOD activity was increased, and kidney weight and renal index were enlarged (Guo Z. et al., 2025). Although some of the literature has demonstrated that hypoxanthine can be used to construct HUA models, most HUA modeling is currently done using a combination of increasing uric acid production and inhibiting its excretion, and there is a relative paucity of literature on modeling with hypoxanthine alone. Usually, other substances (e.g., potassium oxonate, adenine, etc.) are applied in combination to improve the stability and reliability of the model.

##### 3.1.1.3 Construction of yeast induction models

Yeast is rich in purine, and after ingestion, purine metabolism is enhanced *in vivo*, and UA production is increased. Yeast induces XOD activity to increase, accelerating the generation of UA. the increase of UA, on the one hand, affects the function or expression of renal uric acid transporter protein, on the other hand, the high level of UA caused by renal injury, all of which reduces the ability of the kidneys to excrete UA. At the same time, yeast induces increased oxidative stress levels in the body, generating a large number of ROS damage to cells, activating the inflammatory response, activating inflammatory signaling pathways such as the NLRP3 inflammasome and releasing pro-inflammatory factors, which further aggravate tissue damage, affecting the metabolism and excretion of UA, and ultimately leading to the occurrence of HUA. LiG et al. found that the serum levels of 5-aminoimidazole ribonucleotide in the mouse serum were higher than those in the mouse yeast extract paste, and the serum level of UA was lower than those in the kidney. Levels of 5-aminoimidazole ribonucleoside, xanthine, hypoxanthine, and UA increased, adenine and taurine decreased, SOD enzyme activity and liver levels of H_2_O_2_ and MDA increased, and serum levels of betaldehyde and β-D-glucosamine increased, suggesting that the successful induction of HUA was accompanied by an aggravation of oxidative stress and an intensification of renal and joint damage by UA deposition (Li et al., 2025). Extract paste induced hyperuricemia model in mice and found that serum uric acid level was significantly increased from 77 ± 11 μmol/L to 118 ± 23 μmol/L, an increase of 41 μmol/L or 53.2%, and hepatic XOD activity was elevated by about 1.48-fold compared with that of normal mice, suggesting the success of the modeling (Kou et al., 2016). HongF et al. fed high protein diets to male White Rock and AA broiler chickens for 10 weeks and found that the high protein group had significantly elevated serum UA, deformed paw joints, sodium urate crystals in synovial and tissue fluids, and kidney damage. However, the high protein diet did not bring additional side effects and was suitable for modeling HUA (Hong et al., 2020).

##### 3.1.1.4 Construction of the fructose induction model

The model of fructose-induced HUA stems from its metabolic properties. When fructose is metabolized in the liver, small intestine and kidney, fructose kinase consumes a lot of ATP and generates AMP, which is decomposed into hypoxanthine, lowering intracellular phosphorus levels, activating AMP deaminase and prompting XOD activity, accelerating purine metabolism, and promoting the generation of UA, and the metabolite lactic acid will also inhibit renal excretion of UA, thus increasing the risk of HUA (Zhang et al., 2020). ZhangP et al. established a HUA model by giving C57BL/6 male mice oral fructose solution (20, 30, 40 g/kg) for 2 weeks. The results showed that the serum UA level of mice was significantly elevated, the levels of purine synthesis-related metabolites (AMP, adenine, adenosine, hypoxanthine, guanine) in the liver were significantly increased, and the hepatic XOR activity was elevated by about 1.5-fold compared with that of the normal mice, suggesting that fructose can interfere with the hepatic purine synthesis pathway to accelerate the formation of the HUA model (Zhang P. et al., 2022). Zhang et al. successfully established a HUA mouse model by feeding 30% fructose solution for a long period of time. HUA mouse model. The model mice showed elevated serum UA, creatinine, urea nitrogen, and urinary protein levels, as well as renal pathological damage, including tubular hypertrophy and dilatation, glomerular basement membrane thickening, and collagen deposition (Zhang C. et al., 2022). WangY et al. successfully established a HUA model in SD rats by allowing them to drink 10% fructose water for 40 days. The model rats had significantly increased serum UA levels, decreased intestinal uric acid excretion, and decreased ABCG2 expression, suggesting that fructose increases serum UA levels by decreasing extrarenal excretion and decreasing ABCG2 expression (Wang et al., 2017).

#### 3.1.2 Inhibition of uric acid excretion pathways

##### 3.1.2.1 Construction of the ethambutol induction model

Ethambutol’s tubular toxicity independently confounds urate excretion analysis. Ethambutol, as an anti-tuberculosis drug (Abdelaziz et al., 2025), which mainly acts on renal tubular UA transporter proteins (OAT1, OAT3, URAT1, and GLUT9) (Zhu et al., 2017)], leads to an increase in tubular reabsorption of UA by competitively inhibiting UA secretion in renal proximal tubules, which then leads to the *in vivo* aggregation of UA and the consequent increase in serum UA levels (Wu et al., 2017). This process is highly similar to the pathological mechanism of elevated blood UA due to impaired UA excretion in human HUA patients, so the ethambutol-induced model has been widely used to study the mechanism of UA excretion and to evaluate the efficacy of pro-UA excretion drugs. Meanwhile, the nephrotoxicity of ethambutol (Collier et al., 1976) also provides another way for the construction of HUA models. Numerous researchers have skillfully utilized this side effect to successfully construct a HUA model by further affecting the normal excretion of UA through ethambutol-induced renal injury. Liu,C et al. induced a chronic HUA mouse model by gavage of 100 mg/kg adenine and 250 mg/kg ethambutol for 2 weeks, and found that serum UA of the model group of mice was elevated by 1.7157-fold, and the volume of the kidney was The results showed that the serum UA of the mice in the model group was increased by 17,157-fold, the kidney volume was reduced, the color was whitish, and the surface was uneven with particles attached. Renal pathology showed tubulointerstitial damage, including degeneration of renal proximal tubular epithelial cells, uric acid crystals, tubular dilatation, and inflammatory cell infiltration (Liu et al., 2019). SuiX et al. found that after 4 weeks of combined gavage modeling with 200 mg/kg adenine and 250 mg/kg ethambutol, the blood UA level of the model group was increased by about 2.5-fold compared with that of the blank group, and the levels of serum XOD, creatinine and urea nitrogen were also significantly increased. HE staining showed different degrees of degeneration and necrosis of renal tubular epithelial cells, dilatation of tubular lumen, brown crystalline deposition in the tubules, infiltration of large number of inflammatory cells in tubular interstitium, and glomerular injury (Sui et al., 2023). This suggests that prolonged modeling time aggravates renal functional and pathological damage, and that different combinations of dose and administration time have a significant effect on the severity of the model.

##### 3.1.2.2 Construction of the niacin induction model

Niacin, also known as vitamin B3, is a water-soluble vitamin that is converted to niacinamide in the body and is used to treat hyperlipidemia (Kothawade et al., 2021). High doses of niacin can cause HUA (Cruess-Callaghan and FitzGerald, 1966), whose mechanisms include: i) inhibiting renal tubular UA secretion and increasing UA reabsorption, thus reducing UA excretion; ii) affecting purine metabolism, which in turn increases UA production; iii) causing toxicity to the kidneys, damaging renal tubular epithelial cells and further aggravating impaired UA excretion; and iv) triggering a renal inflammatory response, releasing inflammatory factors and activating NLRP3 inflammasomes, thus further aggravate renal injury. EtebariK et al. found that the addition of different concentrations of nicotinamide (1–3 g/L) to mulberry leaves could mimic the effects of vitamin B_3_ overdose on larvae of the silkworm, *Bombyx mori*. The results showed that higher concentrations of nicotinamide resulted in higher levels of UA in the hemolymph of silkworm larvae (0.31 μg/mL at 1 g/L, up to 0.75 μg/mL at 3 g/L, and 0.34 μg/mL in the control group), whereas biochemicals, such as proteins, cholesterol, and triglycerides, were significantly reduced, which suggests that excess nicotinamide interferes with the normal metabolism of silkworm larvae, which in turn affects their growth and development (Etebari and Matindoost, 2004). However, the niacin-induced HUA model suffers from a dose-dependence problem, and a high dose of niacin, although inducing significant HUA, may trigger other side effects. Therefore, the optimal dose range needs to be determined by preexperimentation to minimize the side effects; at the same time, attempts can be made to combine with other HUA inducers to enhance the induction effect and reduce the single drug dose.

#### 3.1.3 Inhibition of uric acid metabolic pathways

##### 3.1.3.1 Construction of the potassium oxonate induction model

Potassium oxalate, because of its structural similarity to UA purine ring, can competitively inhibit the activity of rodent UA enzymes and mimic the impaired UA metabolism in humans, resulting in the accumulation of UA and elevation of blood UA, which is commonly used in the preparation of HUA models for both rats and mice. SuQ et al. induced a rat model of HUA by the consecutive administration of potassium oxalate at 650 mg/kg for 12 weeks, and found that, at 8 and 12 weeks after the administration of the potassium oxalate, the serum uric acid level was significantly higher than that of the normal control group (Su et al., 2018). HanR et al. successfully induced a HUA mouse model by intraperitoneal injection of potassium oxalate at 250 mg/kg/d for 21 consecutive days. The results showed that the model mice had a significant 1.87-fold increase in serum UA levels, a significant decrease in body weight, and an increase in 24-h urine output, blood urea nitrogen and creatinine levels. Renal pathology revealed edema of renal tubular epithelial cells, light cytoplasmic staining and tubular dilatation; intestinal pathology revealed intestinal epithelial detachment, villi breakage and significant reduction in villi number (Han et al., 2025). However, although the administration of the drug alone can be rapid modeling, but the excretion is fast and difficult to maintain for a long period of time, so it is mostly used in the form of co-administration.

##### 3.1.3.2 Construction of multi-drug combination modeling

Currently, the relatively mature and widely used animal models of HUA are the combination of several different modeling drugs, commonly a two-combination, i.e., the combination of UA precursors, UOX inhibitors, or inhibitors of UA excretion, to play a synergistic effect of elevating UA. This type of combination modeling is mainly achieved through the mechanisms of increasing UA production, inhibiting UA excretion, and inhibiting UA metabolism. The most widely used combinations include potassium oxonate + hypoxanthine, adenine + potassium oxonate or ethambutol, and potassium oxonate + yeast. Among them, the combination of potassium oxonate, by inhibiting uricase activity, and hypoxanthine, by increasing uric acid production, significantly increased blood UA levels and stabilized the model. As QiX et al. found that the HUA model was induced in rats by intraperitoneal injection of 500 mg/kg potassium oxonate and 500 mg/kg hypoxanthine for seven consecutive days, the results showed that serum UA, creatinine, and urea nitrogen levels were significantly elevated in the model group of rats, whereas the urinary UA, urinary creatinine, and uric acid excretion fractions were significantly decreased (Qi et al., 2023). Adenine inhibits the renal excretory capacity of UA by converting to water-insoluble dihydroxyadenine under the action of XOD and accumulating in renal tubules, while potassium oxonate inhibits uric enzyme activity and further reduces the metabolism of UA. WangL et al. found that by gavage of mice with adenine (50 mg/kg) and potassium oxonate (300 mg/kg) for 14 consecutive days, compared with the normal control group, mice in the model group had significantly higher serum UA, urea nitrogen and creatinine levels, significant glomerular and tubular damage, increased blue-stained areas in renal tissues, and aggravated fibrosis (Wang et al., 2024). The combination of adenine, which increases UA production, and ethambutol, which inhibits UA excretion, can significantly increase blood UA levels. LiN et al. found that rats in the model group had significantly higher serum UA, creatinine, and urea nitrogen levels by gavage of a mixture of 100 mg/kg adenine and 250 mg/kg ethambutol hydrochloride for 3 weeks, and that there was significant pathological damage to the kidneys, manifested as glomerular sclerosis, tubulointerstitial fibrosis, small renal arterioles sclerosis, crystalline deposits were seen in the renal papillary collecting tubules, and the renal interstitium had an inflammatory reaction (Li N. et al., 2022). Yeast is rich in purines, which can increase UA production; adenine further increases blood UA levels by inhibiting the kidney’s ability to excrete uric acid. ZengY et al. successfully established a model of HUA by gavaging ICR mice with 20 g/kg/day of yeast extract for 21 consecutive days and intraperitoneally injecting 300 mg/kg/day of potassium oxazepam 4 h after gavage. The results showed that the serum UA, creatinine and urea nitrogen levels were elevated in the model group mice, and the kidneys showed obvious pathological damage, which manifested as glomerular atrophy, tubular epithelial cell swelling, and renal interstitial fibrosis (Zeng et al., 2020).

While widely adopted, multi-drug models require strict standardization of drug ratios, administration routes, and strain-specific dosing to ensure inter-laboratory reproducibility. Recent validation studies confirm that adherence to published protocols minimizes variability, with consistent hyperuricemic induction achieved across independent laboratories when critical parameters are maintained.

### 3.2 Gene editing models

“While systemic knockouts (e.g., UOX KO) face embryonic lethality, tissue-specific models (e.g., hepatic UOX KO) achieve stable hyperuricemia without mortality. Unintended consequences—such as metabolic syndrome in GLUT9 KO mice—are not mere artifacts but clinically relevant comorbidities, mirroring human disease synergies. Translational utility is evidenced by URAT1 KO mice validating uricosurics (e.g., dotinurad), where systemic effects are pharmacologically separable.

#### 3.2.1 Construction of UOX knockout model

The UOX gene was mutated in human evolution, resulting in a loss of function of UA enzyme and causing significantly higher serum UA levels in humans than in other mammals. Rodents have a normal Uox gene that degrades UA to allantoin. To construct a model of HUA, researchers knocked out the mouse Uox gene, causing it to lose the ability to degrade uric acid, thus mimicking human HUA (Li H. et al., 2022). As GuoY et al. researchers used the Uox knockout (KO) mouse model for HUA modeling, they found that serum UA levels in KO mice were significantly higher than those in wild-type mice (9.51 ± 0.46 mg/dL and 2.55 ± 0.33 mg/dL, respectively). In addition, KO mice showed impaired intestinal epithelial barrier function and decreased relative abundance of beneficial bacteria in the intestinal tract, especially short-chain fatty acids (SCFAs, e.g., acetic acid, propionic acid, and butyric acid)-producing bacteria, which led to a significant reduction in the concentration of these beneficial metabolites (Guo et al., 2021). PangL et al. constructed a liver-specific Uox knockout mouse model using the Cre/loxP gene targeting system. HUA model was successfully established. The results showed that the serum UA levels in the model group were significantly higher than those in the control group (395.6 μmol/L and 102 μmol/L, respectively). Meanwhile, Uox knockout mice showed significant changes in serum metabolites related to UA metabolism (e.g., UA, hypoxanthine, and xanthine) and a significant increase in hepatic purine metabolites (e.g., IMP, AMP, and GMP), which indicated that the *de novo* purine synthesis pathway was activated in the liver. In addition, the model mice showed pathological changes such as renal tubular atrophy, interstitial inflammation and increased renal fibrosis (Pang et al., 2024). Given the high similarity between the characteristics of UA-affected renal injury demonstrated by this model and the chronic renal injury presented by long-term untreated HUA patients, it can be regarded as a key model for exploring the pathological mechanisms of HUA in the developmental progression of chronic kidney disease, but it still suffers from the shortcomings of high embryonic lethality and susceptibility to renal injury and pancreatic islet cell damage, which in turn affects glucose tolerance.

#### 3.2.2 Construction of UA transporter knockout models

##### 3.2.2.1 GLUT9 gene knockout

Glucose transporter protein 9 (GLUT9) is a high-capacity uric acid transporter protein that is mainly expressed in the liver, intestine, and kidney, and sequence mutations in GLUT9 can lead to HUA in humans (Ruiz et al., 2018). Currently, knockout studies for GLUT9 protein include systemic knockout (G9KO), liver-specific knockout (LG9KO), intestinal cell-specific knockout (G9EKO) and kidney-specific knockout (kiko). Among them, G9KO mice showed impaired UA reabsorption, significant increase in serum UA concentration, increase in water intake and urine output, and further renal pathological damage such as tubulointerstitial nephritis, which is similar to the features of human UA salt nephropathy. LüscherBP et al. found that in the proximal tubules of the kidney, GLUT9 reabsorbed UA into the blood at the basolateral membrane, using the G9KO mouse model. In addition, GLUT9a and GLUT9b were localized in the microvillous membrane of the placental syncytial trophoblast. In G9KO fetuses, serum UA concentrations were significantly higher than in the mother, 4.95-fold under normal diet and 4.10-fold under inosine supplementation. This suggests that the placenta efficiently transports UA from the fetal-placental unit to the maternal circulation via the GLUT9 transport system (Lüscher et al., 2022a). LüscherBP’s team found, using the LG9KO mouse model, that maternal HUA during gestation caused a significant elevation of blood pressure in mid-to late-gestation, with a difference in blood pressure of up to 20 mmHg at the time of delivery, and loss of circadian rhythmicity of blood pressure in late-gestation, with macrophage infiltration in the kidney. The G9KO model showed that fetal HUA resulted in UA levels that were 5.3 times higher than those of control fetuses and 5 times higher than those of their mothers, postnatal growth restriction in female offspring, more pronounced growth restriction in male offspring in a high UA environment, and severe renal injury manifesting as pyelonephrosis, tubular atrophy, and interstitial fibrosis, which were further exacerbated by an inosine diet (Lüscher et al., 2022b).

Serum UA levels were higher in LG9KO mice than in G9KO mice, which may be attributed to the relative elevation of serum UA due to increased reabsorption of urate by GLUT9 in the kidneys. PreitnerF et al. successfully induced HUA (blood UA levels of up to 500 µM) and acute renal injury by intraperitoneal injection of hypoxanthine over a period of 3 days in high-fat chow-fed LG9KO mice (blood creatinine levels were significantly elevated fivefold), and renal pathological changes and inflammatory responses such as tubular dilatation, crystalline deposition and increased expression of pro-inflammatory cytokines were observed (Preitner et al., 2013). The G9EKO mouse model constructed by DeBoschBJ et al. successfully mimicked early stage HUA due to the intestinal cell-specific deficiency of GLUT9, which impaired uric acid uptake and efflux, and significantly elevated serum and urinary UA concentration And metabolic syndrome features such as obesity, dyslipidemia, insulin resistance, hypertension, hepatic fat deposition and cardiac hypertrophy appeared, which can be used to study the role of HUA in metabolic syndrome and its complications (DeBosch et al., 2014). AubersonM et al. constructed a kiKO mouse model and specifically knocked down GLUT9 in the proximal and distal tubules of the kidney, and found that UA/creatinine values and urine volume in their urine increased, with hyper-UAuria and polyuria, but normal plasma UA levels and renal structure. It is speculated that this phenomenon is related to the expression of UA enzymes, partial knockdown of hepatic GLUT9 and potential UA salt transporter compensatory mechanism in mice, suggesting that the knockdown method has limitations of precision and needs to be improved and optimized (Auberson et al., 2018).

##### 3.2.2.2 URAT1 gene knockout

URAT1, a uric acid transporter located on the brush border membrane of renal tubular epithelial cells, is a key factor in maintaining serum UA levels and is primarily responsible for approximately 90% of UA reabsorption. Recent studies have shown that dual-target XOD/URAT1 inhibitors are effective in ameliorating HUA (Zheng et al., 2024), while piperine can ameliorate HUA nephropathy by inhibiting the URAT1/GLUT9 and AKT-mTOR pathways (Li L. et al., 2024). The URAT1 gene knockout mouse model uses gene editing technology to knock out or mutate the key regions of the URAT1 gene, thereby disrupting the function of the uric acid transporter encoded by it. Under normal physiological conditions, URAT1 is mainly responsible for the reabsorption of uric acid in the proximal convoluted tubules of the kidney and the stability of plasma UA levels. When the URAT1 gene is knocked out, the reabsorption capacity of the renal proximal convoluted tubule to UA is significantly reduced, resulting in a large amount of UA being excreted in the urine, resulting in HUA. The excretion rate of UA in mice in this model is significantly increased, while plasma UA levels may decrease or remain the same, depending on the compensatory effects of other UA transporters. In addition, due to the accumulation of a large amount of UA in the renal tubules, it may lead to kidney stone formation and kidney damage. Cai W et al. found that hURAT1-KI mice successfully expressed hURAT1 protein at the apex of the proximal tubular epithelium of the kidney, while the native hURAT1 was located in the human kidney. After further hypoxanthine challenge, the blood UA level in hURAT1-KI mice increased to 251 μmol/L, an increase of approximately 37% compared to 183.5 μmol/L in wild-type mice (Cai et al., 2025). The URAT1 knockout mouse model can not only be used to study the molecular mechanisms of UA metabolism and excretion, but also as an important tool to evaluate the efficacy of drugs for the treatment of HUA and gout (Song et al., 2021). Through this model, we can gain insight into the key role of URAT1 in UA metabolism and the potential application of its inhibitors in the treatment of HUA.

##### 3.2.2.3 ABCG2 gene knockout

ABCG2 is a protein with high-volume urate transport activity, which is widely expressed in various tissues such as the liver, kidney, and intestine, and is responsible for UA excretion, and its dysfunction can increase the risk of HUA (Hoque et al., 2020). It was found that ABCG2 was expressed in the villi and crypts of all intestinal segments in male SD rats, and the ABCG2 content and XOR activity were higher in duodenum and jejunum. In the HUA model rats, the content of ABCG2 in the ileum was significantly increased, and the expression of ABCG2 in the villi and crypts was upregulated, while the other intestinal segments remained unchanged (Morimoto et al., 2020). After the knockout of ABCG2 gene, intestinal UA excretion is impaired, UA accumulates in the body, the renal excretion burden increases, and when its excretion capacity is saturated, the hematuria UA level increases. Takada T et al. found that the HUA model was constructed by treating male Abcg2 knockout mice with 2.0% potassium oxazinate for more than 1 week. The results showed that compared with wild-type mice, the serum UA, renal UA excretion, and UA clearance/creatinine clearance ratio were significantly increased, the renal UA excretion was increased, and the intestinal UA excretion was reduced to less than half, while the bile uric acid excretion was not significantly different (Takada et al., 2014). This is consistent with the findings of Ichida K et al., which showed that Abcg2 knockout mice showed features such as elevated serum UA levels, increased renal UA salt excretion, and decreased intestinal UA salt excretion (Ic et al., 2012). At present, in the process of constructing HUA animal models, GLUT9, URAT1 and ABCG2 gene knockouts usually need to be combined with chemical drug induction to ensure the stability of the models. However, there were significant differences in the effects of different gene knockouts on the survival rate and serum UA level of the model. Gene-edited systems circumvent nephrotoxic confounders, offering superior fidelity for urate mechanism studies.

### 3.3 Construction of environmental factor modeling

The high temperature and high humidity environment is an important cause of HUA (Helget and Mikuls, 2022). In this environment, the body’s core body temperature rises and the heat load increases, leading to profuse sweating and water loss, which in turn leads to urine concentration, decreased UA excretion, and ultimately an increase in blood UA levels (Liu et al., 2021). At the same time, high temperature may directly affect UA excretion function of the kidneys. Cheng Yaxin et al. used a high-temperature and high-humidity incubator to establish a HUA rat model. On the basis of potassium oxazinate or high-purine diet, rats were placed in an incubator at 37 °C ± 0.5 °C, 80% ± 1% humidity, and exposed for 1 h daily. The results showed that the blood UA level of rats in the high temperature and high humidity group and the high purine diet combined with high temperature and high humidity group was significantly higher than that in the other groups. Renal pathology showed that the proximal convoluted tubular epithelial cells were swollen, the lumen was narrowed, and the renal tubulointerstitium was mildly fibrotic, and the deposition of UA salt crystals, podocyte swelling and partial foot process fusion were seen under electron microscopy, and the number of mitochondria and lysosomes in renal tubular epithelial cells increased. At the same time, the level of UA reabsorption transporter URAT1 in the kidney was significantly increased, while the expression of the ABCG2 transporter associated with UA excretion decreased, suggesting that environmental factors can lead to an increase in serum UA levels by promoting UA reabsorption and inhibiting its excretion (Cheng et al., 2023). Although there has been a correlation between ambient temperature and humidity and blood UA levels (Schlader et al., 2019), there is a relative lack of research on specific animal experiments to demonstrate this factor. Therefore, future studies should further explore the long-term effects of high temperature and high humidity on HUA, the combined effects of multiple environmental factors, molecular mechanisms, and interventions, so as to better understand the role of environmental factors in the occurrence and development of HUA.

### 3.4 Construction of motion modeling

The exercise intervention model was constructed based on the effect of exercise on uric acid metabolism. Lactic acid produced by high-intensity exercise competes with uric acid for excretion pathways, while muscle contraction increases adenine nucleotide catabolism, producing more uric acid precursors, resulting in an increase in blood uric acid levels. In addition, high-intensity exercise is also involved in skeletal muscle metabolism and antioxidant protection. In contrast, low- and moderate-intensity exercise has a small effect on UA levels and may even help reduce blood UA levels (Hou et al., 2021). Moderate aerobic exercise is thought to be beneficial in reducing serum UA levels, while high-intensity or short-term acute exercise may lead to elevated serum UA (Zhang et al., 2024). Lollo et al. first adapted the rats to the treadmill (10 m/min, 10 min), and then performed high-intensity exercise tests according to the time-speedometer (1–90 min, 15 m/min, 91–100 min, 20 m/min, 150 min to exhaustion 22 m/min), and found that after exercise, the blood UA level of rats increased significantly, the neutrophil count in the immune system indicators increased, the monocyte and lymphocyte counts decreased, and the total number of white blood cells decreased, and the HDL-C level in the blood lipid index increased. Decreased TAG levels suggest that high-intensity exercise promotes UA production (Lollo et al., 2012). According to Daniela et al., after 12 weeks of training, plasma UA levels increased significantly from (11.80 ± 1.60) μmol/L at rest to (14.45 ± 2.43) μmol/L (an increase of about 22%) after 12 weeks of training and remained at (14.66 ± 1.22) μmol/L (an increase of about 24%) after 30 min. This suggests that exercise has an immediate and sustained effect on UA metabolism (Alberghina et al., 2011). However, this model is rarely used in the study of HUA animal models, because it faces challenges such as allowing experimental animals to cooperate with exercise, controlling exercise intensity and time, and has poor stability and large individual differences, which may affect the experimental results.

### 3.5 Construction of intestinal microecological modeling

Disruption of the intestinal microbiota is a common method for constructing HUA models, including the use of broad-spectrum antibiotics and a high-purine diet. Antibiotics disrupt the balance of intestinal flora and affect uric acid metabolism. Liu X et al. successfully induced HUA by feeding 6-week-old male Wistar rats with yeast-containing diet and gavage with 100 mg/kg adenine for 5 weeks, supplemented with antibiotics (ampicillin 250 mg/kg, neomycin 250 mg/kg, metronidazole 50 mg/kg). The results showed that the serum levels of UA, urea nitrogen, creatinine and total cholesterol in HUA rats were significantly increased, and the relative abundance of Bacteroidites in the gut microbiota decreased, while the relative abundance of Firmicutes and Actinobacteria increased. In addition, the recipient rats were transplanted with fecal bacteria in 1 mL of PBS homogenate in 1 mL of PBS, and UA content of the recipient rats in the third week after transplantation was significantly higher than that in the recipient rats that received normal rat fecal transplantation, confirming the important role of gut microbiota in HUA (Liu et al., 2020). Sun X et al. successfully established a HUA rat model by a high-purine diet (AIN-93M feed mixed with yeast 4:1). The results showed that the serum activities of UA, adenosine deaminase and XOD in the model group were significantly higher than those in the control group, indicating the success of the model. Gut microbiota analysis showed that the diversity of the model group increased (Ace and Chao indices increased, Simpson index decreased), *Bacteroides* and Actinobacteria increased, Firmicutes decreased, and specific genera such as Norank-f-Muribaculaceae increased, and Coriobacteriaceae and *Helicobacter* increased significantly. Correlation analysis showed that ADA, XOD, and UA levels were significantly correlated with gut microbiota composition (Sun et al., 2022). Given that intestinal anaerobes play an important role in UA metabolism, anti-anaerobic antibiotics may be used as a more accurate modeling tool to further explore the independent role of intestinal microbiota in the pathogenesis of HUA. Critically, dysbiosis-induced hyperuricemia is distinguished from antibiotic toxicity by (1) deploying anaerobic-specific antibiotics (e.g., metronidazole): that spare aerobic commensals and reduce inflammation; and (2) FMT causality testing—transfer of hyperuricemic microbiota elevates serum UA in naïve recipients by >40%, while UA normalization after targeted probiotics (e.g., *Lactobacillus* spp.) confirms microbiota-driven pathology.

## 4 Evaluation system of animal models

**FIGURE 3:**
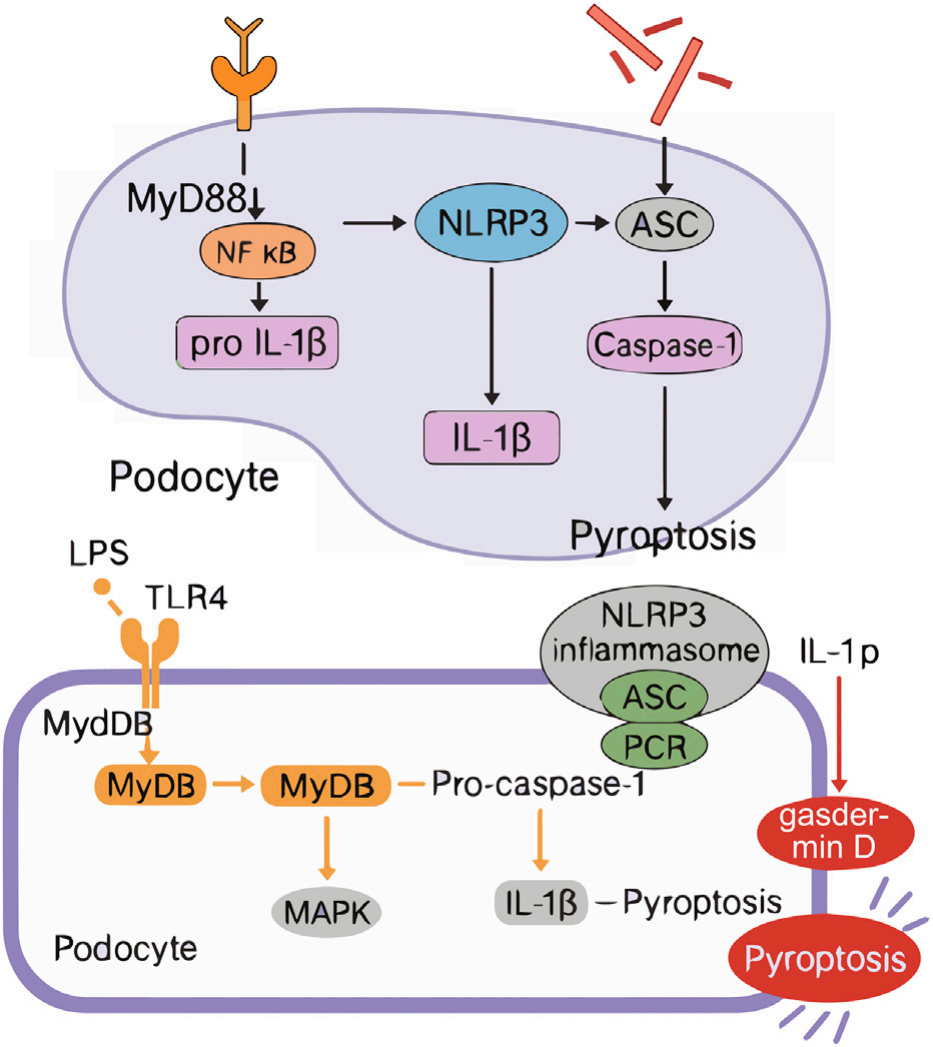
Integrated Workflow of HUA Animal Model Selection and TLR4/NLRP3 Signaling Mechanism in Podocyte Py-roptosis. ASC (Apoptosis-associated speck-like protein containing a CARD), GSDMD (Gasdermin D), TLR4 (Toll-like receptor 4), MyD88 (Myeloid differentiation primary response 88), NF-k B (Nuclear factor kappa B), NLRP3 (NLR family pyrin domain containing 3), IL-1ẞ (Inter- leukin-1 beta), IL-18 (Interleukin-18).

Animal models are a key tool in biomedical research to unravel disease mechanisms and drive the development of new therapies and diagnostic technologies to improve human and animal health (Hoenerhoff et al., 2021). However, there are a wide variety of assays used to evaluate the success of HUA animal models, and there is a lack of unified standards. Through data mining and analysis of 281 HUA animal models, Qiu Guangnan et al. found that 98.22% of the studies detected biochemical indicators, among which the frequency of blood UA detection was the highest (97.15%), which was the key indicator of the success of modeling. Urea nitrogen (51.60%), creatinine (51.96%), XOD (52.67%), triglycerides (41.64%) and total cholesterol (34.87%) were also commonly used indicators. The detection frequency of pathological indicators was 48.75%, which was mainly used to directly observe the degree of damage to tissues and organs, including assessing the morphological changes and weight changes of liver and kidney, and observing whether there was urate crystal deposition and inflammatory response in the joint cavity. Epimetric measures were evaluated at a frequency of 18.15 percent and were primarily used to assess clinical manifestations in animal models, such as ankle swelling (to assess inflammation by measuring ankle circumference) and body weight measurements (to assess the animal’s overall health and disease progression), but are rarely used due to their subjectivity (Qiu et al., 2023). Therefore, we believe that the increase in serum UA level combined with renal pathological injury is the best model detection indicator for HUA. The significant increase in serum UA level is the core feature of HUA, which directly reflects the abnormal state of UA metabolism *in vivo* and is a key biochemical marker to judge the success of modeling. At the same time, the degree of pathological damage of the kidney, as the main organ excreted by UA, can intuitively reflect the substantial damage of HUA to the body, including the lesions of renal tubules and glomeruli, urate crystal deposition, etc. The combination of the two can not only confirm the metabolic disorder of UA from the biochemical level, but also intuitively assess the organ damage from the histopathological perspective, so as to more comprehensively reflect the pathophysiological process of HUA. This comprehensive detection method can effectively avoid the misjudgment that may be caused by a single index, improve the accuracy and reliability of the successful evaluation of modeling, and provide a more reliable model basis for subsequent drug screening and mechanism research. This protocol systematically stratifies biomarkers into three progressive tiers: essential, mechanistic, and pathological. The foundational Tier one requires concurrent measurement of serum uric acid (UA) levels and species-specific metabolites—such as urinary allantoin in uricase-expressing rodents or crystalline deposits in poultry—to confirm hyperuricemia while accounting for metabolic divergence. Tier 2 advances to mechanistic validation through quantitative analysis of UA transporter expression (renal URAT1/GLUT9 and intestinal ABCG2) and rate-limiting enzymatic activity (hepatic xanthine oxidase, XOD), directly addressing genetic and physiological variations. Finally, Tier 3 evaluates end-organ damage via standardized histopathological scoring of UA-mediated injuries, including renal tubular atrophy, urate crystal deposition, and inflammatory infiltration. Beyond serum UA and renal histopathology, comprehensive model validation now requires multi-parametric biomarker profiling: inflammatory cytokines (IL-1β, TNF-α) to quantify NLRP3 inflammasome activation; renal/intestinal transporter expression (URAT1, GLUT9, ABCG2) via qPCR or immunohistochemistry to confirm excretion defects; and oxidative stress markers (MDA, SOD) reflecting UA-induced cellular damage. This triad resolves model-specific phenotypes—e.g., distinguishing pure metabolic hyperuricemia from inflammatory subtypes—and aligns endpoints with human disease mechanisms. Figure 3 illustrates the integrated workflow for hyperuricemia model selection alongside the key TLR4/NLRP3-mediated signaling pathways driving podocyte pyroptosis.

## 5 Discussion

Guided by our proposed three-dimensional framework—which maps animal models across mechanistic (urate overproduction, impaired excretion, inflammatory sequelae), methodological (chemical, genetic, environmental/microbiota), and translational axes (drug validation, target discovery, biomarker development)—this review synthesizes key advances in hyperuricemia modeling. This integrative approach clarifies model selection rationale and uncovers synergistic opportunities, such as combining gene-edited and environmental strategies to address clinical complexities. The HUA animal model provides a key experimental tool for the study of the pathological mechanism of the disease and lays the foundation for the exploration of drug development and treatment strategies. The ideal HUA animal model should be able to accurately mimic the pathological characteristics of human disease, be highly stable and reproducible, and have a high correlation with human disease. In this paper, we systematically summarize the latest research progress in the screening, construction and evaluation system of HUA animal models.

The selection of animal models is central to experimental design. Rodents, such as rats and mice, are the most commonly used models in HUA research due to their rapid reproduction, low cost, ease of management, and similar metabolic properties to humans. However, the presence of UA enzyme genes in rodents is significantly different from the lack of UA enzyme metabolism in humans, so it is often necessary to inhibit UA enzyme activity by chemical drugs or combine with other chemical agents to construct models. Poultry animals (such as chickens and quails) lack UA enzyme genes, and their purine metabolism end product UA is highly similar to human metabolism characteristics, which is suitable for inducing HUA model by high-purine diet, but its physiological structure is quite different from that of mammals, and gene editing technology is relatively lagging behind. Emerging models such as primates, zebrafish, and silkworms have shown unique advantages in HUA research, but their application is still in its infancy due to factors such as high feeding costs, long reproductive cycles, and ethical constraints. Although rodents are widely used in HUA research, they have natural drawbacks. The UA levels in rodents are significantly different from those in humans, and the diagnosis of HUA in humans is not based on a single UA level exceeding the standard, but on the continuous increase in blood UA levels over a certain period of time. However, some studies have not explored the long-term elevation of blood UA levels in rodents, suggesting that the current model can only simulate a certain stage of HUA, but it is difficult to fully reproduce the pathological process of human HUA. Therefore, rodent models alone may not be able to fully simulate the complex pathological features of human HUA.

Critically, these models have propelled therapeutic innovation: oxonate-hypoxanthine models validated clinical xanthine oxidase inhibitors, while humanized URAT1-KI mice confirmed uricosurics including dotinurad prior to FDA approval. Gene-edited ABCG2 KO systems exposed intestinal urate excretion as a target, accelerating drugs like tranilast into trials. Microbiota dysbiosis models further identified probiotics (*Lactobacillus* gasseri) that reduce purine uptake in humans. This synergy underscores the models’ translational power in bridging mechanistic insights to clinical applications.

In terms of model construction, it can be divided into five categories: chemical drug induction, gene editing, environmental factors, exercise intervention, and intestinal microecological regulation. Chemical drug induction is currently the most widely used method, including increasing uric acid sources (such as adenine, hypoxanthine, yeast, fructose, etc.), inhibiting uric acid excretion (such as ethambutol, niacin, etc.), inhibiting uric acid metabolism (such as potassium oxyzinate), and multi-drug combined modeling (such as potassium oxazinate, hypoxanthine, adenine, potassium oxyazinate, or ethambutol, potassium oxazinate, yeast). These methods promote uric acid accumulation by increasing uric acid production, inhibiting excretion, and the body’s own metabolism, which can lead to HUA and even urate nephropathy (HN). Multi-drug combined modeling is favored by researchers because it can synergistically increase blood UA levels through multiple mechanisms and make up for the instability of the dose of a single modeling drug. However, chemically induced models lack UOX or uric acid transporter and are more suitable for drug screening and application research.

The gene editing model constructs a HUA model by knocking out genes related to uric acid metabolism (such as UOX, GLUT9, URAT1, ABCG2, etc.), which can accurately simulate the genetic characteristics of human HUA. However, there are some limitations to this type of model. Mice with Uox gene knockout alone are susgiven to diabetes due to severe kidney damage and pancreatic cell β cell death (Lu et al., 2020); URAT1-Uox double-knockout mice have been used as experimental models for renal hypoUA and exercise-induced acute kidney injury (Hosoyamada et al., 2016). The GLUT9 knockout model showed the characteristics of metabolic syndrome. The ABCG2 gene is expressed in excretory organs such as the liver, kidneys, and small intestine, and also has the function of protecting the brain, testes, and fetus from foreign substances. In addition, ABCG2 is expressed in lactating mammary glands in mice, sheep, dairy cows, and humans (Vlaming et al., 2009).

Although current studies have not explored the potential for unstable damage in these organs and tissues due to ABCG2 gene knockout, it can be speculated that its knockout may have a potential impact on the function of these organs. The most puzzling thing is that the serum UA concentrations of knockout mice vary greatly between groups, which makes it difficult to compare multiple models. In addition, there is a difference in the distribution and expression of UA salt transporters in rodents and humans, and UA enzyme-encoding gene UOX is silent in humans (Roman, 2023), but it is expressed in multiple tissues of rodents (Gustin et al., 2010). The GLUT9 gene is expressed in the basolateral membrane of the proximal tubule of human kidney (Augustin et al., 2004), but in mice, it is expressed in multiple organs, mainly localized to the liver, intestine and kidney. URAT1 is predominantly expressed in the brush-like border membrane of proximal convoluted tubular epithelial cells of the human kidney (Guo W. et al., 2025). In mice, the mouse homologous gene URAT1 is predominantly expressed in liver and brown adipose tissue. Studies have shown that deletion of URAT1 exacerbates acetaminophen-induced liver injury in mice (Zhao et al., 2025), aggravates hepatic steatosis in mice, and causes brown adipose tissue to turn white, thereby promoting the development of insulin resistance (Tanaka et al., 2022). The ABCG2 gene is predominantly expressed in the human small intestine, and in zebrafish, ABCG2a is the only homolog expressed in the blood-brain barrier (BBB) of adult and juvenile fish, and its expression is localized in the claudin-5-positive cerebrovascular system (Thomas et al., 2024). Given these differences, gene-editing models need to be further optimized to more accurately mimic human disease states.

The incidence of HUA is influenced by a combination of environmental and genetic factors, and is closely related to lifestyle. The HUA model induced by high temperature and humidity environment, exercise intervention and intestinal microecology has obvious advantages, but there are few relevant research literature, and the stability of the model needs to be further verified. In addition, the current evaluation of HUA animal models mainly relies on serum UA level, which is a single indicator, and there is a lack of unified evaluation criteria. Some studies only examined the levels of biochemical indicators such as UA and creatinine, which is difficult to fully reflect the complex pathophysiological process of HUA. In fact, the pathogenesis of human HUA involves multiple factors, so the combined modeling method is usually used to simulate various pathogenic conditions and risk factors, but the complex pathogenesis of human HUA cannot be fully reproduced.

As for the evaluation system of HUA, we found that the combined modeling method can also be applied to the study of HN, and its modeling time and dosage are basically the same as those of HUA (Qian et al., 2025). This raises the question of whether there is an over-molding. Whether the basic experiment is dominated by HUA or its complications (such as kidney disease) can be analyzed whether there is a constant indicator to analyze whether the model only exists in the HUA stage and does not progress to the stage of kidney disease? In addition, HUA and HN are treated differently, and there may be a risk of overtreatment if the patient is treated for kidney disease before the stage of kidney disease.

This review establishes that effective hyperuricemia modeling requires pathophysiological alignment with human disease subtypes: chemical induction offers rapid screening utility but suffers from nephrotoxic confounders; gene-edited systems enable precise mechanism dissection despite translational limitations; and environmental/microbiota models best replicate clinical comorbidities. Critically, the proposed tiered evaluation framework (integrating serum UA, transporter quantification, and NLRP3-associated inflammation markers) addresses standardization gaps across species. Future efforts must prioritize humanized rodent models (e.g., UOX KO + hURAT1 KI) to bridge species divergence, multi-omics validation of gut microbiota-urate crosstalk, and longitudinal designs capturing chronic progression from hyperuricemia to end-organ damage. While animal models remain indispensable for elucidating the complex pathophysiology of hyperuricemia (HUA) and evaluating systemic therapeutic effects, the growing emphasis on the 3Rs/4Rs principle (Reduction, Refinement, Replacement, and Responsibility) in biomedical research necessitates a critical evaluation of *in vitro* approaches. Integrating these alternatives not only addresses ethical imperatives but can also enhance the efficiency and translatability of HUA research by complementing animal studies, particularly in early screening and mechanistic dissection phases. These directions will accelerate therapeutic translation beyond current symptom management.

Recent pharmacological efforts extend beyond xanthine oxidase inhibition to include agents that directly modulate pyroptotic pathways and urate transport. Small-molecule NLRP3 inhibitors (e.g., MCC950) and gasdermin D blockers have shown efficacy in animal models by preventing inflammasome-driven podocyte death and preserving renal function. Meanwhile, novel uricosuric drugs targeting URAT1 and ABCG2, as well as dual-action compounds that combine XO inhibition with urate transporter modulation, are under preclinical investigation and have improved outcomes in hyperuricemic rodents. Adjunctive therapies such as SIRT1 activators or probiotics that upregulate ABCG2 expression and suppress NLRP3 activation further illustrate how integrated targeting of metabolic and inflammatory axes may offer superior protection against HUA-associated renal injury. Beyond pharmacologic agents, non-pharmacologic strategies show growing promise in preclinical and clinical settings. Dietary interventions—particularly low-purine diets and reduced fructose intake—directly limit uric acid precursors, while specific probiotics (e.g., *Lactobacillus* gasseri PA-3) modulate gut microbiota to reduce purine absorption and enhance intestinal urate excretion via ABCG2 upregulation. Exercise regimens also require careful calibration: moderate aerobic activity lowers serum UA by improving renal function and insulin sensitivity, whereas high-intensity exercise may transiently elevate UA via lactate competition and ATP catabolism. These approaches, when combined with pharmacotherapy, offer synergistic benefits for holistic hyperuricemia management.
